# Patient and public involvement (PPI) in prisons: the involvement of people living in prison in the research process – a systematic scoping review

**DOI:** 10.1186/s40352-021-00154-6

**Published:** 2021-11-11

**Authors:** Samantha Treacy, Steven Martin, Nelum Samarutilake, Tine Van Bortel

**Affiliations:** 1 Hilary Rodham Clinton School of Law, Swansea University, Swansea, UK; 2 Cambridge Public Health, Department of Psychiatry, School of Clinical Medicine, University of Cambridge, Cambridge, UK; 3 Leicester School of Allied Health Sciences, De Montfort University, Leicester, UK

**Keywords:** Patient and public involvement in research, Engagement in research, Participatory research, Prison research, People living in prison, People formerly living in prison, Prisoners, Ex-prisoners, Scoping review

## Abstract

**Background:**

Patient and Public Involvement (PPI) in health and social care research is increasingly prevalent and is promoted in policy as a means of improving the validity of research. This also applies to people living in prison and using social care services. Whilst evidence for the effectiveness of PPI was limited and reviews of its application in prisons were not found, the infancy of the evidence base and moral and ethical reasons for involvement mean that PPI continues to be advocated in the community and in prisons.

**Objectives:**

To conduct a review of the literature regarding the involvement of people or persons living in prison (PLiP) in health and social care research focused on: (i) aims; (ii) types of involvement; (iii) evaluations and findings; (iv) barriers and solutions; and (v) feasibility of undertaking a systematic review.

**Methods:**

A systematic scoping review was undertaken following Arksey and O’Malley’s (International Journal of Social Research Methodology 8: 19-32, 2005) five-stage framework. A comprehensive search was conducted involving ten electronic databases up until December 2020 using patient involvement and context related search terms. A review-specific spreadsheet was created following the PICO formula, and a narrative synthesis approach was taken to answer the research questions. PRISMA guidelines were followed in reporting.

**Results:**

39 papers were selected for inclusion in the review. The majority of these took a ‘participatory’ approach to prisoner involvement, which occurred at most stages during the research process except for more ‘higher’ level research operations (funding applications and project management), and only one study was led by PLiPs. Few studies involved an evaluation of the involvement of PLiP, and this was mostly PLiP or researcher reflections without formal or independent analysis, and largely reported a positive impact. Barriers to the involvement of PLiP coalesced around power differences and prison bureaucracy.

**Conclusion:**

Given the very high risk of bias arising from the available ‘evaluations’, it was not possible to derive firm conclusions about the effectiveness of PLiP involvement in the research process. In addition, given the state of the evidence base, it was felt that a systematic review would not be feasible until more evaluations were undertaken using a range of methodologies to develop the field further.

**Supplementary Information:**

The online version contains supplementary material available at 10.1186/s40352-021-00154-6.

## Background

Patient and Public Involvement (PPI) has been defined as “research being carried out **‘**with**’** or ‘by’ members of the public rather than ‘to’, ‘about’ or ‘for’ them” (INVOLVE, 2012). This has been promoted in UK government policy (Health Research Authority, 2017), is supported by their major research funding body the *National Institute for Health Services Research* (Russell et al., 2019), and national support centre *INVOLVE*, and has become increasingly prevalent in the UK over the last decade (Ball et al., 2019).

The involvement of patients or the public in research is rooted in social justice and disability rights activism (Beresford, 2002; Glassman & Erdem, 2014; Macauley, 2017), and aims to move from paternalistic to democratic practices in health and social care with knowledge and expertise shared between ‘professionals’ and ‘patients’ leading to better quality and more valid research (Aubin et al., 2019; Hoekstra et al., 2020; Madden & Speed, 2017). However, the systematic, rapid evidence and literature reviews that have been conducted on PPI in health and social care research have indicated that there is typically a lack of evidence of effectiveness (Samele et al., 2008; Treacy et al., 2019). Although positive impacts on patients, research quality, the research team and research system were widely reported (Ball et al., 2019; Brett et al., 2012), conclusions were severely limited as the research was generally of poor quality, with much of the ‘evidence’ based on researchers’ perceptions rather than on robust evaluation (Aubin et al., 2019; Ball et al., 2019; Cook et al., 2019; Mockford et al., 2012).

In addition, a number of issues were documented that reportedly impeded implementation including: a lack of definition or theoretical basis guiding involvement (Mockford et al., 2012), increased costs and resources (Domecq et al., 2014), differences of opinion regarding the ‘representativeness’ of the patients and its necessity (Lander et al., 2019; Morgan, 2016), and tokenistic involvement (Madden & Speed, 2017; Ocloo & Matthews, 2016). Despite this, much of the literature maintained that PPI arose largely for moral and ethical reasons, which may have resulted in an evidence lag and advocated better evaluations and a focus on overcoming cultural challenges to ensure better implementation (Aubin et al., 2019; Ward, Bailey & Boyd, 2012).

This paper specifically reviews the literature on the involvement of PLiP in health and social care research, which is also supported by policy on the involvement of ‘the public’ in research (Health Research Authority, 2017). For the purposes of this paper, PLiPs include people incarcerated in both prisons and jails. The general health of PLiP is typically reported to be poorer than that of people living in the community possibly due to more chaotic lifestyles including alcohol and substance misuse, less access to healthcare and stress related to the ‘pains’ of imprisonment (Crewe, 2011; Williams et al., 2012). The sharp rise in older PLiP numbers over the last few decades (Lee et al, 2016; Sturge, 2020) has also, inevitably, brought with it an exponential increase in health and social care needs with an estimated 85% of PLiPs aged over 50 reporting problems (Lee et al, 2019; Hayes et al., 2012; Senior, Forsyth, Walsh et al., 2013). Whilst policy and legislation promotes equivalence between the community and prisons regarding health and social care (Care Act, 2014; Department of Health, 1999), a recent parliamentary inquiry concluded that the government is “failing in its duty of care” to PLiPs (Health and Social Care Committee, 2018). In addition, PLiPs’ health and social care issues have reportedly been exacerbated by the restricted regimes and services and suspension of visits applied in prisons in response to the Covid-19 pandemic (Brennan, 2020; Her Majesty’s Prison & Probation Service, 2020), the impact of which have left PLiP and their families in “extreme distress and desperation”, particularly those most vulnerable (Prison Reform Trust, 2020, p. 7; Clarke, 2020).

It is unclear how equivalent PLiP involvement in the health and social care research process is relative to that in the community as no reviews of the literature were found. However, PPI in prisons has been described as under-developed (Awenat et al., 2017). A study of prison and research staffs’ attitudes towards PLiP involvement demonstrated a lack of understanding and suggested that PLiPs were not seen as potential partners in the process (Johnson et al., 2018). One review outlined the possible ways that PLiPs could be involved, referring to the involvement of forensic mental health service users in research, but no examples of practice in prison were presented (Samele et al., 2008).

Given the extent of PLiP needs and their position as perhaps the most disempowered health and social care service users, their involvement in the research process would be highly consistent with the moral and ethical concerns driving public involvement in research (Revolving Doors Agency, 2016). Therefore, it is the aim of this paper to explore the literature for examples of PLiP involvement in the research process and to undertake a systematic scoping review addressing the following research questions:

1. What were the aims of the research which involved PLiPs or people who were formerly living in prison (PFLiP) during the research process?
2. In what ways were PLiPs and PFLiPs involved in the research process?
3. Was PLiP or PFLiP involvement evaluated, and how was this carried out? What were the main findings of these evaluations?
4. What were the main obstacles in involving PLiPs or PFLiPs, and what were the suggested solutions for overcoming them?
5. Would it be feasible to undertake a systematic review of PLiP or PFLiP involvement in health and social care research?

## Methods

Given the lack of reviews of PLiP involvement in health and social care research, a scoping review methodology was considered the most appropriate to explore the aims of the study. Scoping reviews are considered to be particularly useful to systematically scope, chart and synthesise the available evidence in a research field with a view to examining whether a full systematic review could be conducted (Munn et al., 2018). This review used the five-stage scoping review framework posited by Arksey and O’Malley (2005), with reporting guided by the PRISMA extension for scoping reviews checklist and explanation (Tricco et al., 2018) – the completed checklist for this review is available in Additional file 1. The five-stage framework followed in this review were:

***Stage 1: Identification of the research question(s) –*** identified in the ‘Background’ section

***Stage 2: Identification of relevant reports – the literature search***

A search strategy to identify review-relevant reports was formulated by the research team, informed by systematic reviews undertaken in relation to patient and public involvement in health and social care research more broadly (Brett et al., 2010; Staley, 2009). Searching included electronic and hand searching components. The electronic database search involved searching ten databases which focused on health and criminal justice, which were: Applied Social Sciences Index and Abstract (ASSIA), Criminal Justice Abstracts, Embase, Medline (OVID), National Criminal Justice Reference Service (NCJRS), Psycinfo, Pubmed, Social Services Abstracts, Sociological Abstracts, and Web of Science (WoS). The search combined research involvement-related terms AND context-related ones (see Table 1), with no date or language restrictions, and covered the full range of publications up until 12th April 2019, with a second updated search from that date until 10th December 2020. Additional file 2 has an example of the search strategy used.

**Table 1:** Search terms used in electronic database search

Research involvement-related terms	Context-related terms
PPI OR PPIE OR “Patient and Public Involvement” OR “prisoner advisor*” OR “participatory health research” OR “participatory action research” OR “participatory research” OR “community based participatory research” OR “service user advisory group” OR “peer research*” OR “advisory committee*” OR “emancipatory research” OR “user(engagement OR involvement OR participation OR representation)” OR “patient(engagement OR involvement OR participation OR representation)” OR “prisoner(engagement OR involvement OR participation OR representation)” OR “offender(engagement OR involvement OR participation OR representation)”	Prison* OR Jail* OR Correctional* OR Penitentiar* OR Penal

The electronic search was supplemented by a comprehensive hand-search, which involved reference mining, and searches using search engines, specialist prisoner researcher journals and PPI-specific journals, online resources or libraries related to prison(er)s or PPI, recommendations from academic networking sites, and directly contacting authors in the field.

***Stage 3: Study selection – inclusion and exclusion criteria***

Papers were selected for inclusion in this review if the following criteria were met informed by the People, Intervention, Comparator and Outcomes (PICO) formula (Richardson et al., 1995):

(i) People: The research process has to involve people who are, or have been, in prison, which includes research conducted by prisoner-led organisations. The research process cannot include prison staff or any other individuals who have not served time in prison.
(ii) Intervention (general): The overall research study has to focus on a health-related topic (including mental or physical health, public health or substance misuse), or be conducted with a health-related professional such as a mental health professional, a general practitioner (physician) and more. Studies that focus on other prison-focused topics (such as education or regime) with no health focus will be excluded.
(iii) Intervention (specific): The study will need to involve people currently in prison or those who have been in prison in the research process itself, and describe the type of involvement. This ‘involvement’ can be from any approach or theoretical tradition and can take any form. If PLiP or PFLiP’s involvement in the research is as ‘participant’ or ‘subject’ only, the study will be excluded.
(iv) Comparator: Any or none. No papers will be excluded on this basis.
(v) Outcomes: No papers will be excluded on the basis of this measure type; the review will chart whether there was an evaluation of PLiP involvement and the outcomes reported. The outcomes of the overall studies will not be reported.
(vi) Context: The overall study has to be set in prison(s) and jails. If the overall health-related study is set in the community, or any other institution, it will be excluded.
(vii) Other criteria (language): Include studies in English, French or Dutch. Exclude papers in all other languages.
(viii) Other criteria (paper type): Only include ‘original’ empirical research of any design or methodology, and literature reviews. Exclude: commentaries, opinion pieces, newspaper and blog articles, conference abstracts and presentations.

Each of the papers was screened by title and abstract by one researcher, and full-text screening was undertaken by two independent researchers and compared to check for inter-rater reliability (Rutter et al., 2010). Differences of opinion were discussed by the researchers and resolved between them. Papers were thus included if they presented health research in prison with PLiP or PFLiP undertaking any research activities as part of the study.

***Stage 4: Charting the data – data extraction***

One researcher extracted data from all of the papers selected, with a second independent researcher extracting data from a third of the papers to check for consistency. Where differences arose, they were resolved between the researchers and consensus reached. Both researchers used an extraction template informed by the PICO formula, and included: author, date, country, overall study aim, aim of prisoner involvement, type of PPI, sample description (if applicable), type of PLiP involved (serving or ex-prisoner), type of prison study situated in, PPI evaluation (whether and how), PPI outcomes on PLiP and research(er), obstacles and recommendations made for PLiP involvement. In addition, there was a section that required extracting data on the research activities which PLiP and PFLiP were involved in. This section combined and followed the categories of research activity suggested by INVOLVE (adapted by Jacobs et al., 2017) and the THIS Institute (Marjanovic et al., 2019), which were conceptualisation, design, fund/commission, undertaking research, analysis, dissemination, evaluation, and implementation. The activity categories were added to when other research-related activity recurred in a number of the papers.

***Stage 5: Collating, summarising and reporting results – data analysis***

The nature of the papers selected for this review precluded any form of meta-analytic synthesis. The focus was on charting the involvement approaches and activities as described in the papers, and a narrative approach was undertaken to synthesise the findings where reported (Popay et al., 2006) as these were wholly qualitative.

## Results

**Fig. 1:**
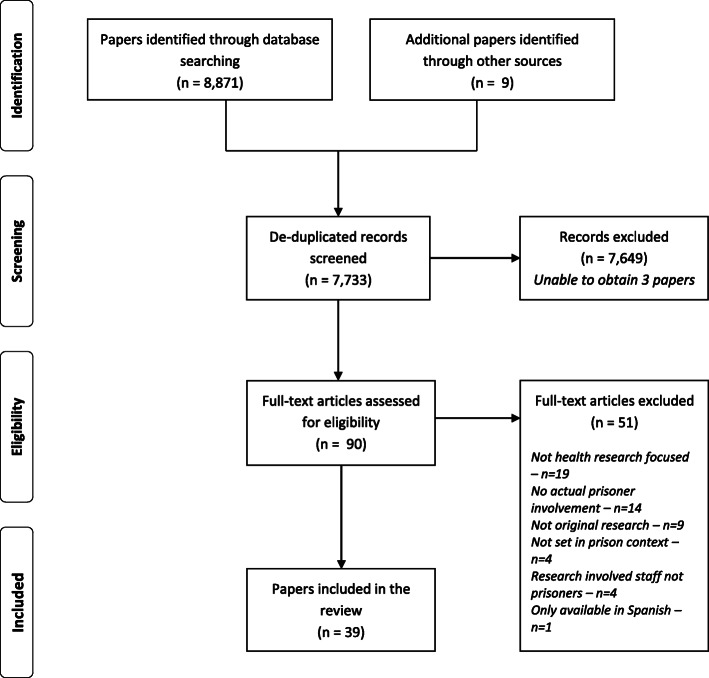
PRISMA flow diagram

The literature search returned 8880 papers of which 7733 remained after duplicates were removed. Following screening, thirty-nine papers were selected for inclusion in this scoping review, having met the requisite review criteria. The stages of the screening process undertaken to select the papers are depicted in Fig. 1.

An overview of the included papers is given in Table 2, which is split between papers that used a broadly participatory approach to PLiP/PFLiP involvement (*n* = 24), and papers that used other approaches (*n* = 14). One review of the literature was also included. The table presents the key features of each paper relating to PLiP/PFLiP involvement as well as evaluation details, main findings and recommendations, where reported.

**Table 2:** Study features

Study No	Author; Year; Country	Study aim	PPI aim or PPI paper aim	PPI type	Type of prisoner	Sample size & type	Prison type	Evaluation & type	Outcomes (i) Prisoners (ii) Research(ers)	(i) Obstacles & (ii) recommendations
(i) ***Participatory Approaches***										
1	Buchanan et al., 2011; Canada	To under-stand PLiPs reasons for substance misuse	Social justice, collaboration, increase sense of control	Participatory research; peer researcher in health research team	PLiPs & PFLiPs	*n* = 88, female, similar to prisoner population, reportedly	Female, min/medium security prison	No	None reported	None reported
2	Crabtree, Ohm et al.; Crabtree, Ohm, et al., 2016 USA	To evaluate an occupati-onal therapy programme	Not reported	PAR model; met 3–4 times per month	PLiPs	n = 3, male	Male minimum security prison	No	(i) None reported; (ii) reflected on potential bias in analysis (academic & prisoner)	(i) bureaucracy affected prisoner availability; (ii) none reported
3	Crabtree, Wall et al.; Crabtree, Wall, & Ohm, 2016; USA	To evaluate an occup-ational therapy programme	Reflective account of PPI, benefits & challenges	PAR model; met 3–4 times per month	PLiPs	n = 3, male, all had college degrees & had worked as clerks	Male minimum security prison	Yes; critical reflection;	(i) an opportunity, helped make sense of prison experience – a ‘palliative’; respect, dignity & relationships important; emancipatory; (ii) PAR team as ‘perfect storm’; mutual learning; significant personal & professional impact; furthered cause of ‘occupational justice’	(i) power skewed; unable to do internet-based research; bureaucracy affected prisoner & room availability; (ii) helps if have pre-existing trust; minimum security prisons; ex-prisoners for analysis; use in-house messaging system to communicate; more participation; important to see change of feel like a system stooge.
4	Curd et al.; Curd et al., 2007; USA	Evaluation of wellness intervention in substance abuse programme	To ensure the programme success & sustain-ability	CBPR model – wellness committee, evaluation advisory panel (EAP)	PLiPs	n = 2 on committee; n = 6–8 in EAP, male	Male min security prison – TC	No	(i) sense of ownership; (ii) developed a more valid intervention (socially & scientifically), involvement in administration relieved staff burden	(i) staff and prisoner turnover, hierarchy, scarce resources, poor data systems; (ii) get staff buy-in, especially seniors; involve in administration to relieve staff burden; turnover can be an opportunity to refresh if some older members remain;
5	Fields et al.; Fields et al., 2008; USA	To reflect on a sexuality focused programme	Foreground prisoner views; determine PAR feasibility; increase understanding & validity	PAR - workshops with education & research	PLiPs, & at least one PFLiP	*n* = 74, female, average age 36 years (range 19–63), priority to women of colour; paid	Female county jail, CA	Yes; reflected on process throughout	(i) increased confidence, compassion empathy, respect, optimism; felt valued; explore own life; use intelligence; (ii) PAR in prison a “particularly liberatory act” (pg 80); roles over-lapped & changed; mutual learning; researchers & institutions all contribute to female prisoner issues (exploit or insensitive); less about health issue & more of relationships, & inequality of race & poverty; never gained full trust – equality elusive; improved the service	(i) power differences; bureaucracy affecting prisoner availability & interrupting process; difficult to promote sexual expression in oppressive system; voluntary attendance may not be possible; (ii) training; design research so women can dip in & out but still contribute (cyclical); opportunities for paid researcher roles on release;
6	Hatton & Fisher; Hatton & Fisher, 2011; USA	To explore the impact of prison & copayment of fees, on health	To describe how PPI facilitated understanding	CBPR – formed community advisory board (CAB)	PFLiPs	N = 3, CAB members	Female prison	No	(i) strengthened case of prison advocates; (ii) increase credibility of research to prisoners; prisoners as bridge in communications with prisoners; vital in analysis; builds knowledge & evidence	(i) only included ex-prisoners as minimised further research obstacles & staff retribution; (ii) help if have positive pre-existing relationship; use of ex-prisoners and confidentiality certificates
7	Hatton et al.; Hatton et al., 2006; USA	To explore prisoner health problems & care	Not reported	Participatory research in collaboration with an NGO including ex-prisoners	PFLiPs from local NGO	Not reported	Female county prison, Western USA	No	None reported	(i) none reported; (ii) training on focus group facilitation
8	Kendall et al., Kendall et al., 2020, Australia	Explore experiences of accessing healthcare and limitations of ‘equal treatment’	Ensure research & community priorities mesh; use valid concepts, establish an advisory group	Community collaborative participatory action research	PLiPs	Forty-three Aboriginal women	Urban and regional prisons in NSW	No	None reported	None reported
9	Martin, Adamson et al.; Martin et al., 2013 Canada	To evaluate the pilot of a fitness programme	To design, lead & evaluate	PAR –participatory research team led by prisoner	PLiPs	Not reported, female	Female, min/medium security prison	No	(i) researchers working in community on related work, post-release; (ii) resultant programme designed & implemented by prisoners, a strength	None reported
10	Martin, Korchinski et al.; Martin et al., 2017; Canada	Team reflection on prisoner participa-tion	PPI involved collabora-tion across the whole project	PAR – research team prisoner-led	PLiPs & PFLiPs	Not reported	Female provincial prison	Yes, reflection	(i) healing/transformative, giving back; continued with interventions post-release; (ii) change can happen when collaborate; altered personally & professionally; new knowledge & relationships; mutual learning; introduced spiritual health as a factor;	(i) research ended when prison warden retired; (ii) need for senior staff buy-in
11	Martin, Murphy, Chan et al.; Martin, Murphy, Chan, et al., 2009; Canada	To explore prisoners’ health issues	Feasibility of doing PAR in prison; research design & conduct	CBPR & transformative action research team	PLiPs	*n* = 120 at a group event, female	Female, min/medium security prison	No	(i&ii) authors reported it was healing & transformative for all; (ii) feasible for prisoners to do research; mutual learning; many questions and possible interventions suggested, prison elders can ‘control’ groups better	(i) none reported; (ii) training in transcription; importance of warden support
12	Martin, Murphy, Hanson, et al., 2009; Canada	To explore prisoner’s health issues & goals	Description of a PAR process; design & develop research	Participatory action research team	PLiPs & PFLiPs	*n* = 190 across the project life span, female	Female, min/ medium security provincial prison	Yes, reflect-ion & quali-tative analysis	(i)meaningful, supportive; increased hope, confidence, communication & transferable skills; altered perspective; (ii) prison as a good place to do participatory work; challenged reductionist conceptions of health – more holistic, ideas larger in scope; learn process by doing	(i) lack of staff buy-in; lack of funding; prisoner turnover; (ii) trained to transcribe; emphasise values and targets in common between staff and research – especially senior; helpful to have a research assistant sustain focus in midst of prisoner changes
13	Martin, Turner et al.; Martin et al., 2018; Canada	The feasibility of implementing HIV prevention in prison	Feasibility of research; Views on future projects, build capacity & partnership	Community-Based Research (CBR)	PLiPs & PFLiPs	n = 12; 3 Aboriginal, average age 50 years (range 30–65)	Male, medium security federal prison, West Canada	No	(i)increase empathy & sharing, helping others; (ii) participatory work feasible; prisoner involvement increased uptake of unpopular service	(i) prisoners lack trust; (ii) if subject is important enough, men will engage; combining professional and prisoner-led sessions; use of tutor with personal experience of topic
14	McLeod et al., McLeod et al., 2020, Canada	Describe a peer health mentoring program for released women	Create social action; improve quality of life	Participatory health research framework	PFLiPs	340 women	Correctional Facility for Women, Canada	No	None reported	None reported
15	Meyer & Fels; Meyer & Fels, 2009; Canada	To explore prisoner health issues	Reflection on the project; focus on analysis	PAR - team	PLiPs	Not reported, female	Female prison	No	(i)reportedly empowered; transferable skills; (ii) better understanding to analysis – that which is not understandable outside; impacts on roles and way they research see institutions; women know what they need	(i) power differences; bias towards researcher interpretation; project closed when warden left; (ii) support of prison warden; follow prisoners when they alter focus – allow to change scope & focus, and be ‘other’; listen; prison staff to hear prisoner stories; not about teaching women, but engaging
16	Perrett et al., Perrett & Gray, 2020, UK	To report on work undertaken with PLiPs on health & wellbeing, and process of peer research	Explore feasibility of PLiPs as peer researchers as part of educational initiative	Participatory Action Research (PAR)	PLiPs	154 men on the vulnerable prisoners unit	Long stay private prison in Wales, UK	Yes, reflection from one peer researcher only	(i) Establishment of the project, data collation and direct communication with management empowering; enabled access training and education, learning new skills; altered power balance; interesting and motivating (ii) findings more representative; facilitated all-prison approach to wellbeing; senior management made aware of issues	(i) process was logistically complicated; focus groups negatively affected by staffing and regime changes (inc. cancellations, need for escorts); limited access to IT causing lots of difficulty; (ii) build in impact evaluation from the start; good prison staff-project staff communication needed; staff buy-in key at all stages, especially management support
17	Ramsden et al.; Ramsden et al., 2015; Canada	To explore ways to improve prisoner health & wellbeing	An analysis of prisoner’s writing	CBPR & transformative action research; peer researchers - team - team	PLiPs & PFLiPs	*n* = 200 approx; 39% < 30 years	Female, min/ medium security prison	Yes, reflect-ion	(i)reportedly transformative & empowering; transferable skills; sense of purpose; optimistic; planning futures; (ii)shared knowledge valued by prison community & contributors	(i) none reported; (ii) feedback on research to prison & contributors – creates relationships; involvement became a work placement, so could be paid; prisoners allowed internet access- could contribute to literature reviews
18	Sherwood & Kendall, 2013; Australia	To explore the ‘Social & Cultural Resilience & Emotional Wellbeing of Aboriginal Mothers in Prison’ project	Description of the participatory approach; feedback on research material	Community Collaborative PAR model	PFLiPs	Not reported, all prisoners Aboriginal	n = 3; all prisons in New South Wales holding women	No	None reported	None reported
19	Sullivan et al., 2008; UK	To explore relapse prevention; evaluate relapse prevention course	Prisoner-led campaign, assisted by researcher	PAR – member of action research group: Breaking the Chain’	PLiPs	n = 8; all male, all wing drug represent-tatives	HMP Grendon: Cat B male prison, wings run as TCs	Yes, reflection	(i) enjoyed success & process; increased confidence, caring, empowerment; opinions valued; but, anger & disillusionment when staff not interested in findings; (ii) have knowledge of system; just a group member; difficult when group angry, but other group members helped group move on	(i) power differences; lack of staff interest; computer access, security issues & unreasonable internal deadlines limited prisoner authorship; (ii) initially academic researchers may need to take a more facilitative and administrative role; (ii) get staff buy-in; negotiate security & access issues at each prison locally
20	Townsend Townsend, 2001; Malaysia	To explore ways to improve care for people who are HIV+	To generate ideas to improve care & support	Participatory assessment process, Participatory Learning & Action tools – formed groups	PLiPs	Prison 1: n = 8; female; Prison 2: n = 12, male, Prison 3: *n* = 30, male; all HIV+ status	N = 3; Prison1: female prison; Prisons 2 & 3: male prisons	No	(i) stimulating, diverting, prestigious; (ii) trust, rapport, motivates; representativeness of prisoners only semi-so; group autonomy important; participatory process key, but “a degree of methodological compromise will probably be inevitable” (pg 10)	(i) prison needs, rather than prisoners’, dictated the research; inter- and intra-group dynamics can negatively impact process; researcher access limited; bureaucracy limits; (ii) de-emphasise goals of social change to officials; manage group dynamics; prisoner groups need to become autonomous quickly; compress research activity as much as possible; manage prisoner expectations; do not curtail freedoms
21	Ward & Bailey, 2011; UK	To develop a self-harm training package for staff	To identify staff training needs, and ways to address them	PAR	PLiPs	Mapping (n = 9); group (n = 16–20); interviews (*n* = 15); surveys (*n* = 50); female; mean age 36 yrs. (range 18–58)	Female prison, England	No	(i) hope there were benefits in effecting change; (ii) key in identifying knowledge gaps; PAR important if trying to have an equivalence between healthcare research in the community and prison	(i) prisoners involved may not be representative – although not a significant issues; (ii) none reported
22	Ward & Bailey, 2012; UK	To develop self-harm care pathways	Examine ethical dilemmas of the research	PAR	PLiPs	n = 2, female	Female prison, England	Yes. Reflection	(i) change should directly benefit; optimism, confidence, insight, good to help others & share; concerns re privacy allayed; empowering, gives agency; (ii) positive experience; PAR as good way to develop services	(i) bureaucracy affects access; potential for ‘vicarious’ trauma; coercion problematic; dilemma of payment; can’t offer full confidentiality; budget cuts de-prioritised research; (ii) get unescorted prisoner access; supervision & occupational health access; balance needs & compromise – pick battles that are most important to prisoners and staff; take time with informed consent; transparency in reporting
23	Ward & Bailey, 2013; UK	To develop self-harm care pathways	To identify service gaps & staff training needs	PAR	PLiPs	Mapping (n = 9); group (n = 16–20); interviews (n = 15); questionnaires (n = 50); female; mean age 36 years (range 18–58)	Female prison, England	No	(i)reportedly empowering; (ii) prisoners take a more holistic approach; PAR can create change and work as well as in the community, despite lack of policy support	(i) power differences led to not training prisoners as researchers; (ii) find other ways to involve prisoners than trained researchers
24	Ward, Bailey & Boyd; Ward & Bailey, 2012; UK	To improve outcomes for people who self-harm	To identify needs regarding staff training	PAR	PLiPs	Not reported, female	Female prison, England	Yes, one prisoners’ reflections	(i)increased confidence, insight; altered staff behaviour positively; positive to see changes, blurred prisoner-staff divide; (ii) PAR possibly beneficial to staff & prisoners	(i) power differences; and concerns about ‘teaching’ staff as a potential problem; (ii) none reported
(ii) ***Other approaches***										
25	Antoniou et al., Antoniou et al., 2019, Canada	To identify the main barriers to engaging with HIV-related medical & social care post-release	To inform programme design, and give people a voice in research and programme	Concept mapping	PFLiPs with HIV	39 participants	n/a	No	(i) Not reported (ii) Analysis occurred in real-time with concrete recommendations rather than later and researcher only; findings informed development of a programme for formerly incarcerated people with HIV.	None reported
26	Apa et al.; Apa et al., 2012; USA	To explore risk factors for spread of staph infection	To get feedback & increase support for the study	No approach reported	PLiPs	Not reported	n = 2, max security, female & male	No	(i) none reported; (ii) collaboration with prisoner groups can increase study support	(i) none reported; (ii) useful to work with groups that represent prisoners in prison
27	Awenat et al.; Awenat et al., 2017; UK	RCT of suicide prevention therapy	To examine prisoners’ experience; to improve study’s ‘ecological validity’	Based on INVOLVE model; monthly Service User Research Group (SURG) meetings	PFLiPs	n = 4; 2 male, 2 female; “mixed ethnicity”; age range 40–60 years	Male prisons – overall study	Yes. Quali-tative; inter-views, IPA analysis	(i) positive to effect change, share ideas; mutual respect, felt valued, make good of bad & give back, positive change in self-perception & perception by others; impacted desistance; one member reported little impact; (ii) guided study amendments – improved quality; may be more important for ex-prisoners because of stigmatised identities	(i) none reported; (ii) training – six-day Master’s level research module; honorary university contracts and staff cards; positive relationships with academic researcher key; open feeling to meetings so free to share views
28	Byng et al.; Byng et al., 2012; UK	To explore continuity of care, & improvements	To increase access to participants, make research & materials more legible	No approach reported; peer researchers - Offender Research Group	PFLiPs	n = 13; paid	Various-not reported	Yes, critical reflection	(i) some felt valued, more confident & optimistic, less isolated; training led to confidence; (ii) strengthened process – “subtle fusion of ideas throughout” (pg 188)	(i) difficulties setting up research groups in prison; university bureaucracy in hiring ex-prisoners led to some dropping out; difficulty managing group dynamics; (ii) allocate enough time; pay researchers & have a lead; training
29	Cornish et al., 2016; UK	To examine older prisoner experiences of release	Not reported	No approach reported– Older prisoner users’ group	PFLiPs	Not reported	n = 5	No	(i) not reported; (ii) prisoners experiences helpful in exploring the experiences of peers	(i) none reported; (ii) training to conduct interviews
30	Edge et al., Edge et al., 2020, UK	To develop an understanding of equivalence via accounts of PLiPs secondary (hospital) care experience	To ensure methods are acceptable; to clarify terminology	Peer-led research (approach not defined)	PFLiPs from a prison charity	N-45. Focus groups (n = 5) and 1:1 interviews (n = 17)	Five English prisons – male & female.	No	(i)Not reported (ii) Believed participants more empowered & gave more honest accounts; terminology clarified; ensured methods acceptable. But, lack of experience and use of own experiences may ‘lead’ participants	None reported
31	Forsyth et al., 2017; UK	To study the effective-ness of a health & social care assessment & planning tool for older prisoners	Not reported	No approach reported. Action learning group; ex-prisoner co-applicant; trial steering committee members	PLiPs in group; PFLiPs on committee & co-applicant	Group= unreported; co-applicant = 1; committee member = 2	Group, n = 1; n = 10 in overall study - from open to training to high security	No	(i)reportedly valued contributing to change; (ii) valued by team across the study	None reported
32	Forsyth et al., Forsyth et al., 2020, UK	To establish prevalence of dementia and mild cognitive impairment in prisoners in England and Wales and their health and social care needs	To give input to study proposal, and advice throughout; ensure consider needs of older people	PPI	PFLiPs	One PFLiP was a co-applicant; with a further research group of four PFLiPs involved	77 prisons in England and Wales – mixed establishments	No	(i) PFLiPs reportedly welcomed research, believing it to fell a gap and help with service development (ii) Input to research design was useful; input to development of training and intervention considered meaningful	None reported
33	Hassan et al., 2014; UK	To explore prevalence & accept-ability of psycho-tropic medication prescribing	Not reported	No approach reported. Monthly research advisory group	PFLiPs	N = 7 regular member; male & female; all used prison healthcare	n-11 in study; male & female	No	(i)improved confidence, communication & networks; accessed training; (ii) ‘Proved’ possible to have a group of ex-prisoners work across the lifespan of a study advice on system & recruitment invaluable; made research more effective	(i) difficulties recruiting led to delays in project; team changes; skill mix; (ii) access to training; collaborating with departments with PPI experience helped recruitment; have group terms of reference & codes of conduct; explain & clarify everything as differing skills; allocate enough time & resources; have administrative help
34	Howerton et al., 2009; UK	To examine influences on prisoners seeking help for mental health problems on release	Not reported	No approach reported. Collaborated with an NGO’s user group	PFLiPs	Not reported	Male, Cat B prison, South England	No	None reported	None reported
35	South et al., 2014; UK	To systematically review prisoner peer health prog-rammes	Opportunity for dialogue, & application of findings	PPI – expert symposium (lay experts), & listening exercises	PFLiPs: symposium & listening exercise; PLiPs: listening exercise	Symposium: unreported; Listening exercises = 8 per prison approx.; all peers or peer supported	n = 3: Cat B local, high security & female prison; in NW England	No	(i)not reported; (ii) PPI as integral; symposium added context, led to a variety of grey literature & intervention types	(i) unable to have serving prisoners at public meetings, so excluded from symposium; questions around representativeness when prisoners selected by staff; (ii) none reported
36	Taylor et al., 2018; UK	RCT for a mental health intervention	To present ways prisoners involved; to contribute to design & delivery	PPI – peer researchers; fortnight group	PFLiPs	n = 8 – rolling member-ship; male; aged 25–56 years, paid	n/a	Yes, reflect-ion	(i)increased confidence to make change – aided recovery, skills & knowledge; did not feel judged; cared for, valued, purposeful, making good of bad; (ii) ex-prisoners can do & develop research, interventions & theory; focus groups – more engaged; analysis – more depth; intervention more applicable; helped explain purpose better; desk-based researchers had contact with study population; emphasised importance of family – and so new aspect to study	(i) conflict over name/image use when disseminating –acknowledgement v future difficulties; (ii) committed, skilled team; funding, staff & system to pay researchers in cash; staff to maintain team contact including returns to prison; meetings in neutral place, relaxed, inclusive and regular – intensive bursts; rolling membership; end date clear; celebrate success & invite family in; continual feedback; use a peer researcher label on materials; compromise on name/image use; support prisoners’ plans; understand role of peer researchers to challenge.
37	Treacy et al., 2019; UK	To evaluate dementia friendly community approach	To assess need for dementia initiatives; material alterations	PPI	PLiPs	*n* = 46 (prison A = 16; prison B = 30); all male	n = 2,; Cat C sex off-ender & local prisons, male	No	(i) none reported; (ii) prisoner input invaluable in involved study tasks	(i) difficulties involving prisoners living with dementia; (ii) none reported
38	User Voice;, 2016; UK	To explore extent of ‘spice’ use & linked problems	The study was managed and led by a peer-led organisation	Peer-led research, so no approach as such	PFLiPs mostly; PLiPs for some tasks	n = 9; Cat C prisoners, England; geography-ically represent-ative	n/a	No	(i)not reported; (ii) more likely to increase trust, rapport & insights from prisoners if speaking to someone from a similar background	None reported
(iii) ***Review***										
39	Samele et al., 2008; UK	A review of prisoner involvement in mental health research	Not reported	Not reported	Not reported	Not reported	n/a	n/a	(i) none reported; (ii) Essentially, there were no examples of service user involvement in mental health research in prisons, lagging behind community research	(i) possible bias of staff selecting prisoners; bureaucracy affects availability; payment is contentious; power differences; prison & research bureaucracy; (ii) consulting forensic involvement projects, and prisoner councils & service development projects for guidance; need time & resources; staff buy-in and liaison person; negotiate around prisoner selection; not to raise expectations; support to be involved; address issues of confidentiality; provide training – should lead to skills and qualifications; agree roles; educate prison staff

Abbreviations: PLiP=People Living in Prison; PFLiP=People who Formerly Lived in Prison; CBPR = Community-Based Participatory Research; PAR = Participatory Action Research; TC = Therapeutic Community

The majority of the included papers were from the United Kingdom (*n* = 19) or North America (*n* = 17, 10 papers from Canada, 7 from the USA), with two more from Australia and one from Malaysia. It is of note that seven of the Canadian papers were generated around one research programme, and two further papers involved some of the same research team. Sixteen of the papers involved only PLiPs in the research process, fourteen included only PFLiPs, and eight involved both PLiPs and PFLiPs. The review paper did not appear to involve people who were or had been in prison in the review process. The numbers of PLiPs or PFLiPs involved in the papers ranged from two to approximately 200. Whilst sixteen papers did not report the gender identities of the PLiPs or PFLiPs involved, and less reported on other demographic details, twelve papers involved females, seven involved males, and four papers included both males and females in the research process. In terms of payment, three studies reported that PLiPs (*n* = 1, paper 5) and PFLiPs (*n* = 2, papers: 28,36) were paid. A further four papers (papers 9–10,12,17), all from one research programme, described how involvement in the research team became a prison work placement, ultimately resulting in payment at prison work rates.

### Research question one: what were the AIMS of involving people who were or had been in prisons in the research process?

As shown in Table 2, the main aims of PLiP or PFLiP involvement were to make the research more ‘valid’, legible or applicable to prisoners (*n* = 19, papers5,6,8,10,15,18,20,21,23-28,30,32,35–37); to determine the feasibility as well as benefits and challenges of PLiP or PFLiP involvement in the research process overall (*n* = 7, papers 3,5,11-13,16,22); to empower PLiPs or PFLiPs, advance social justice or detail commissioned prisoner-led work (*n* = 8, papers 1,5,9,14,19,25,35,38), and to increase the chances of research success or sustainability (*n* = 3, papers 4,26,28).

### Research question two: in what ways were people who were or had been in prison involved in the research process?

PLiP or PFLiP involvement largely happened within a research-specific group, team or advisory panels, which mostly worked together with academic researchers (*n* = 26, papers 1–6,8-12,14-17,19,20,25,27–33). Six papers also detailed the involvement of PLiPs or PFLiPs on trial or project steering committees (papers 4,6,31-33,38), in an expert symposium and listening exercises (*n* = 1, paper 35); and as a co-applicant on projects (*n* = 2, paper 31,32). One study included was also entirely PFLiP-led and managed research (paper 38).

**Fig. 2:**
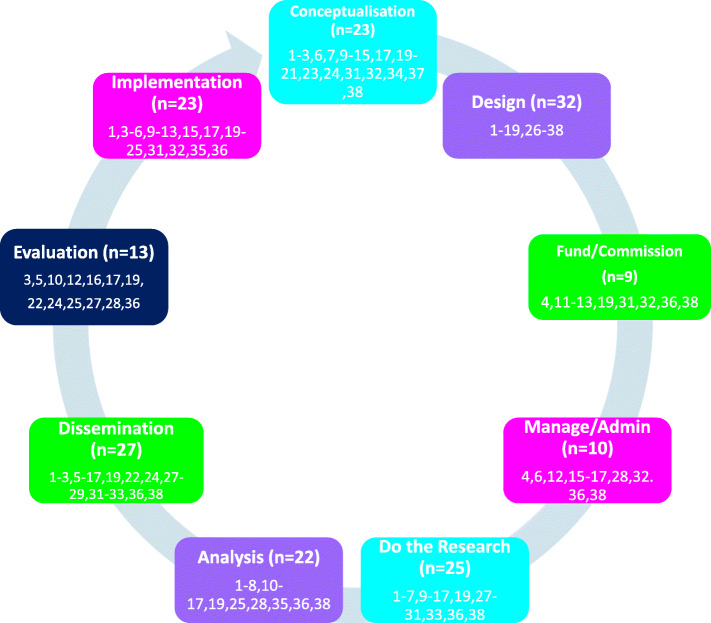
Types of involvement of people living in prison

Figure 2 shows how PLiPs or PFLiPs were involved in each part of the research process. The majority of papers involved PLiP or PFLiPs in generating ideas or, research questions or conceptualising the research and evaluation (*n* = 23). A very high number of papers involved people in research design (*n* = 32), predominantly in co-designing or feeding back on research materials (*n* = 24, papers 1–6,8,11-19,28-30,33,34,36–38), with some studies also reporting involvement in overall research design (*n* = 16, papers 3–5,7-11,16,18,19,27,28,32,33,35). The majority of papers also involved PLiPs or PFLiP in undertaking research (*n* = 25) largely in collecting questionnaires (*n* = 10, papers 1,9-11,13,14,16,17,19,38) or conducting or co-conducting interviews and focus groups (*n* = 14, papers 1–3,5-7,10,16,19,28-30,36,38); and, in dissemination (*n* = 27) with PLiPs or PFLiPs authoring or co-authoring papers (*n* = 22, papers 1–3,5,9-12,14-17,19,22,24,28,29,31-33,36,38), and presenting or co-presenting findings (*n* = 13, papers 3, 5,6,10-12,15-17,19,27,33,36). A number of studies (n = 22) also involved individuals in feeding back views on preliminary findings, or in the interpretation and analysis of qualitative data, with involvement in quantitative analysis explicitly reported by only two studies, one of which was led by PFLiPs (papers 19,38). This was despite the majority of papers employing a mixed methodology (*n* = 22; papers 1,3,4,6,9,12-14,16,18-21,23,24,28,31,32,34,36–38), and a further two using a quantitative approach (papers 26,33). The remaining papers used a qualitative methodology or were a review. A large number of studies also involved PLiPs or PFLiPs feeding into, planning or implementing resultant prison programmes or service changes (*n* = 23).

PLiPs and PFLiPs were less involved in funding or commissioning applications (*n* = 9), with only three papers reporting PLiPs or PFLiPs as co-authoring, or co-applying for funding applications (papers 12,31,36), and two PLiP and PFLiP-led research teams were commissioned to undertake research (papers 19,38). In addition, only ten papers described involvement in research administration or management which mostly included taking responsibility for the functioning of the research team (papers 4,6,12,15,17,36,38), but also in training research staff (paper 36), and interviewing prospective academic researchers (paper 28). Lastly, there were only thirteen papers that detailed the involvement of PLiPs or PFLiPs in project evaluations, which is described further in Research Question Three.

### Research question three: was prisoner involvement evaluated, and how? What were the main findings?

The majority of the papers presented no evaluation of PLiP or PFLiP involvement (*n* = 26), and indeed the included literature review on prison mental health research found no studies that evaluated the involvement of PLiPs or PFLiPs in the research process (paper 39). Of the twelve papers that did present some evaluative detail, this mostly took the form of PLiP or PFLiP (critical) reflections (papers 3,5,10,12,16,17,19,22,24,27,28,36); one paper subjected the reflections to a qualitative analysis (paper 12), and another paper involved interviewing PLiPs and analysing the data qualitatively (paper 27).

The findings of the papers’ ‘evaluations’ were broken down into impact of involvement on (i) the PLiPs and PFLiPs and (ii) the research and researchers. It is of note, that whilst a number of the papers did not present evaluative or reflective detail, some papers did include authors’ observations on impact, which will also be included here. Regarding (i) impact on PLiPs and PFLiPs, the papers mostly reported positive findings in relation to emotional effects – with increased confidence, optimism and empowerment (*n* = 12, papers 5,12,15-17,19,22-24,28,33,36), healing, compassion and giving back (*n* = 9, papers 5,10,11,13,17,19,22,27,36), and understanding and developing a more positive perspective on themselves (*n* = 7, papers 3,5,9,10,20,27,29). It was also reported that PLiPs and PFLiPs found the work meaningful and purposeful (*n* = 6, papers 12,16,17,19,20,36), emancipatory (*n* = 3, papers 3,5,27), that they felt valued and respected (*n* = 6, papers 3,5,19,27,28,36), and that they appreciated the transferable skills and knowledge that they learned (*n* = 6, papers 12,15-17,33,36), and the opportunity to be involved in service change and future research work (*n* = 8, papers 3,5,9,10,20,27,29,32). One paper also suggested that involvement had also increased desistance (paper 27). There were very few negative findings reported, with one paper each respectively reporting: frustration at not being listened to (paper 19), concerns about personal privacy (paper 22), and – more neutrally – that the involvement work had had little impact (paper 27).

The impact of PLiP and PFLiP involvement on (ii) the research process and/or the researchers, largely focused on the process. There were reports that involvement increased understanding and knowledge with PLiP or PFLiPs offering new or more ‘holistic’ insights (*n* = 10, papers 5,6,10-12,15,16,19,21,23), and enhancing the validity, quality or applicability of the research (*n* = 12, papers 4,9,16,25,27,28,30,32,33,35,36,38). In addition, seven papers stated that PLiP work contributed to the success of the research (papers 6,10,13,20,22,26,28), co-created service change (*n* = 8, papers 5,10,13,16,21,23,25,32), and six papers reported that involving PLiP in the research process was feasible (papers 11,13,16,24,33,36). Some of the researchers also reflected on the mutual learning that took place during the process (*n* = 3, papers 3,10,11), the personal transformative impact on them and their perspectives (*n* = 4, papers 3,10,11,22), and the impact on them professionally (n = 4, papers 3,5,10,15), including reflecting on their role and purpose, and of the sometimes negative impact of institutions and norms (which they are part of) on the lives of PLiP. There were only four less positive reflections made on the process: that group dynamics were difficult to manage (*n* = 2, papers 19,28); that conflict arose around using PLiP names and images in project materials – with project staff concerned about how being identified as a PLiP or PFLiP could have a negative impact in future (paper 36); that peer researchers’ lack of experience or use of personal experience may ‘lead’ study participants (paper 30); and that involving PLiPs and PFLiPs in a prison context will always involve methodological compromise (paper 20).

### Research question four: what were the main obstacles and solutions for overcoming them?

A number of obstacles to PLiP or PFLiP involvement in research were described. The most frequently reported was that power differences between PLiPs or PFLiPs and academic researchers – amplified when within a hierarchical prison system – meant that the equality inherent in participatory approaches was even more difficult to attain (*n* = 9, papers 1,3-5,15,19,20,23,24). The impact of prison bureaucracy was also described as a barrier in terms of PLiP availability (*n* = 6, papers: 1,3,5,16,19,20), lack of computer/internet access limiting types of involvement and communication (*n* = 3, papers 3,16,19), room availability and interruptions (*n* = 3, papers: 3,5,16), and limiting researcher access to PLiPs (*n* = 2, papers 20,22). There were also reported barriers to involvement arising from (ex-)PLiP turnover (*n* = 3, papers 4,12,33), lack of funding and resources (*n* = 3, papers: 4,12,22), and a lack of staff belief in the process or research (*n* = 3, papers: 12,16,19). Three papers also specifically detailed the impossibility of involving PLiPs in their research, so much so that they only involved PFLiPs (*n* = 3, papers19,28,35). In addition, researchers described dilemmas in conducting research involving PLiPs around voluntariness (papers 5,22), payment (paper 22) and confidentiality (paper 22), as well as university bureaucracy negatively impacting recruitment (paper 28).

In addition, a number of recommendations for the involvement of PLiP and PFLiPs were also made, which included:

- Emphasise staff buy-in (especially senior staff and overall prison governor or warden), common values and goals.
- Ensure adequate funds for the research and staff time to conduct it.
- Designating an administrative worker for the study.
- Training PLiPs for the role and responsibilities expected of them (e.g. facilitating focus groups).
- Not being overly prescriptive about process where PLiP or PFLiPs lead.
- Regularly share feedback of findings and news.
- Paying researchers for their work (e.g. PLiP involvement could become a work placement to enable this.
- Building evaluation into the research design from the beginning.

## Discussion

### Summary of findings

Most of the included papers used ‘participatory’ approaches, which are typically bottom-up, community-generated and sometimes explicitly political ways of working, alongside the more apolitical, top-down PPI-type approaches. This was broadly consistent with findings in community studies (Hoekstra et al., 2020), although were more defined in the prison literature (Mockford et al., 2012). PLiP involvement was more collaborative than PLiP-led as opposed to PPI in the community studies (Ocloo & Matthews, 2016), with PLiPs and PFLiPs involved in most aspects of the research process, although with less involvement in higher-level operations such as funding applications or commissioned projects, and project administration or management. Very few of the studies included an evaluation. Of those that did, these were largely a presentation of PLiP or PFLiP or researcher reflections not subject to any formal analysis, with mostly positive impacts on PLiPs and PFLiPs, the research process, and the team reported. There were very few negative reports – as with community-based studies (Aubin et al., 2019; Ball et al., 2019; Brett et al., 2012; Cook et al., 2019; Mockford et al., 2012). In terms of the barriers, a number of studies discussed similar issues around involvement activities as those carried out in the community but possibly magnified by the prison setting. These included more entrenched power differences, prison bureaucracy, lack of funding and resources, staff attitudes and ethical concerns. It would appear that these issues, which besiege prison research more generally (Charles et al., 2016; James, 2013; Matfin, 2000), present very particular issues for PLiP and PFLiP involvement in the research process that need careful consideration.

### Addressing research question five: the feasibility of undertaking a systematic review of the involvement of people in prison?

With a lack of robust evaluations, there is obviously a high risk of bias in the articles included in this review and, consistent with reviews of public involvement in the community, this makes it difficult to draw any conclusions from the evidence presented. Given the lack of evidence available or found for this review, this would suggest that conducting a full systematic review of the literature may not be feasible until the evidence base is somewhat stronger than it currently is.

### Review limitations

There were a number of aspects of this review that could limit the extent to which the findings can be applied, which derive from: (i) the papers included, and (ii) the way the review was conducted.

(i) *The papers included*

The majority of papers included were from high income countries. Whilst this may reflect the languages spoken by the review team members, it is also reflective of the “northern epistemic hegemony” (Aas, 2012) apparent in much research. Given that at least some participatory approaches have their roots in the work of Freire (1970) and more emancipatory, anticolonial and political struggles in low and middle-income countries (Glassman & Erdem, 2014; Macauley, 2017), it is likely that at least some prison participatory work is happening that is either unpublished or not captured within the review’s search strategy due to language barriers.

In addition, a number of the papers were drawn from a research group in Canada, who appeared to run a number of linked research projects funded over a longer period of time which would benefit robust relationship building and involvement activities (Buchanan et al., 2011; Kendall et al., 2020; Martin et al., Martin, Murphy, Chan, et al., 2009, Martin et al., 2013, Martin et al., 2017, Martin et al., 2018; Ramsden et al., 2015). Obviously, this may be different for the more short-term funded studies and timeframes that that many prison projects are conducted within, and therefore not be readily generalisable as they may not be reflective of the type and extent of involvement and relationships possible to develop within that shorter timeframe, which may skew some of the findings of the review. That said, there were some shorter-term studies included, and it is hoped that the findings and recommendations of both the shorter and longer-term projects will be useful.

(ii) *The review process*

None of the review team were PLiPs or PFLiPs and, as such, this may limit the analysis. This review was intended as a scoping of the available evidence to assess the feasibility of conducting a full systematic review. Whilst at the present time this would be considered unfeasible, at a later date when the evidence base is more populated, it would be a firm recommendation of this review that the review team would be broadened to include or indeed be led by someone (or team of people) who lives or has lived in prison.

This review also provided no assessment of study quality. Given that there was a lack of evaluations that could be assessed for quality in a more ‘traditional’ sense, this was justified. However, there appears to be a need for some form of specific standard assessment of quality of public involvement, both in the community and in prisons, which does not currently exist (Brett et al., 2010). It may be that using a tool such as GRIPP2 (Staniszewska et al., 2017) to record the ways that PLiP or PFLiP involvement is defined and reported, alongside charting the issues raised in the Public Involvement Impact Assessment Framework (Popay, Collins et al., 2014) in a well-described, systematic way, may be useful.

### Recommendations

Further recommendations, aside from those arising from the review limitations, also arose from the combination of difficulties in public involvement generally and its application in a prison setting more specifically. These are:

- Whilst prison staff buy-in is very important in a prison context and may indeed need some local, regional or national government prison policy support, as no research can successfully occur in prisons without addressing these ‘gate-keepers’ (Matfin, 2000), researcher buy-in is equally important. There is a lack of understanding amongst some researchers of the potentially positive impact of public involvement especially for research quality improvement; hence, education and training at all career stages may be useful (Biggane et al., 2019; Johnson et al., 2018).
- Research in prisons can take a long time to set-up and conduct (Hayes & Senior, 2007), and involving public in research can take even more time and resources, particularly where PLiPs or PFLiPs need additional training (Domecq et al., 2014). Therefore, it is important that this is adequately accounted for in the commissioning and funding of research, and in making funding applications, as it is possible that PLiP involvement could be marginalized.
- Whilst the issues of payment of PLiPs for their involvement may be controversial, it has been possible to include it as a work or education placement in one of the included papers (Fields et al., 2008). This could indeed be possible in prisons in England and Wales but it would need more centralized government support to facilitate it and, similarly, local, regional or national government support in other jurisdictions;
- Evaluations – some researchers have highlighted the complexity of disentangling the relative contributions of patient and professional researchers (Aubin et al., 2019), whilst others have suggested that this is over-complicating the issue (Boivin et al., 2018). Given the embryonic nature of PLiP involvement in the ‘research evidence journey’ (Nutley, Powell & Davies, Nutley et al., 2013), conducting a variety of studies to populate the evidence base ranging from more quantitative and qualitative exploratory studies to more robust evaluations would help to gain a greater understanding of impact, ‘what works’ and ‘what does not work’ in this specific context, and develop solutions to barriers regarding power differences, bureaucracy, cultural issues and ethical concerns.

## Conclusions

A number of the findings of this review are consistent with those of reviews undertaken of patient and public involvement in health and social care research in the community – particularly the lack of evaluations, and therefore lack of evidence of effectiveness. There were also similar barriers to implementation, although somewhat amplified by the hierarchical structure of the prison setting. Importantly, this review highlights that there are a number of studies of PLiP or PFLiP involvement across most aspects of the research cycle. However, there is a lack of involvement in higher-level research operations, and indeed PLiP or PFLiP-led research. It is important that this work is more robustly evaluated in order to not only provide evidence of effectiveness, but to more meaningfully explore challenges to PLiP or PFLiP prisoner involvement and to develop practice in the field. The health and social care needs of PLiPs are manifold, and governments are reluctant to grant early release, even to those in very ill health. It is therefore imperative that this particularly vulnerable and disempowered group of people are given voice in health and social care research in order to potentially improve their situation.

## Supplementary information

**Additional file 1: Appendix 1.** PRISMA-ScR-Fillable-Checklist_09.08.21.

**Additional file 2: Appendix 2.** Example Search Strategy (initial search).

## Data Availability

All data and materials used in this review are included in this article and its appendices.
