# Supplementary material for: Patient and public involvement (PPI) in prisons: the involvement of people living in prison in the research process – a systematic scoping review
Source: Health Justice. 2021 Nov 11;9:30. doi: 10.1186/s40352-021-00154-6 (PMC8584641; doi:10.1186/s40352-021-00154-6)
Supplement: Supplementary file 2 — Additional file 2: Appendix 2. Example Search Strategy (initial search). [file 40352_2021_154_MOESM2_ESM.docx]

**Appendix 2: Example Search Strategy (initial search)**

Database: Ovid MEDLINE(R) and Epub Ahead of Print, In-Process & Other Non-Indexed Citations, Daily and Versions(R) <1946 to April 11, 2019>

Search Strategy:

--------------------------------------------------------------------------------

1 PPI.mp. (15236)

2 PPIE.mp. (44)

3 "patient and public involvement".mp. (483)

4 "prison advisor*".mp. (0)

5 "participatory health research".mp. (46)

6 "participatory action research".mp. or participatory research/ (4710)

7 "community based participatory research".mp. (5061)

8 "service user advisory group".mp. (4)

9 "peer research*".mp. (100)

10 "advisory committee*".mp. (13675)

11 "emancipatory research".mp. (14)

12 1 or 2 or 3 or 4 or 5 or 6 or 7 or 8 or 9 or 10 or 11 (35207)

13 (user adj (engagement or involvement or participation or representation)).mp. [mp=title, abstract, original title, name of substance word, subject heading word, floating sub-heading word, keyword heading word, organism supplementary concept word, protocol supplementary concept word, rare disease supplementary concept word, unique identifier, synonyms] (1304)

14 (patient adj (engagement or involvement or participation or representation)).mp. [mp=title, abstract, original title, name of substance word, subject heading word, floating sub-heading word, keyword heading word, organism supplementary concept word, protocol supplementary concept word, rare disease supplementary concept word, unique identifier, synonyms] (27823)

15 (prisoner adj (engagement or involvement or participation or representation)).mp. [mp=title, abstract, original title, name of substance word, subject heading word, floating sub-heading word, keyword heading word, organism supplementary concept word, protocol supplementary concept word, rare disease supplementary concept word, unique identifier, synonyms] (8)

16 (offender adj (engagement or involvement or participation or representation)).mp. [mp=title, abstract, original title, name of substance word, subject heading word, floating sub-heading word, keyword heading word, organism supplementary concept word, protocol supplementary concept word, rare disease supplementary concept word, unique identifier, synonyms] (1)

17 13 or 14 or 15 or 16 (28869)

18 12 or 17 (63505)

19 prisoner/ or prison/ or prison*.mp. (27237)

20 jail*.mp. (3249)

21 correctional*.mp. (3310)

22 penitentiar*.mp. (590)

23 penal.mp. (1639)

24 19 or 20 or 21 or 22 or 23 (30777)

25 18 and 24 (199)

***************************
